# High performing flexible optoelectronic devices using thin films of topological insulator

**DOI:** 10.1038/s41598-020-80738-8

**Published:** 2021-01-12

**Authors:** Animesh Pandey, Reena Yadav, Mandeep Kaur, Preetam Singh, Anurag Gupta, Sudhir Husale

**Affiliations:** 1 Academy of Scientific and Innovative Research (AcSIR), Council of Scientific and Industrial Research, National Physical Laboratory, Dr. K. S Krishnan Road, New Delhi, 110012 India; 2 Council of Scientific and Industrial Research, National Physical Laboratory, Dr. K. S Krishnan Road, New Delhi, 110012 India

**Keywords:** Materials science, Nanoscience and technology, Optics and photonics, Physics

## Abstract

Topological insulators (TIs) possess exciting nonlinear optical properties due to presence of metallic surface states with the Dirac fermions and are predicted as a promising material for broadspectral phodotection ranging from UV (ultraviolet) to deep IR (infrared) or terahertz range. The recent experimental reports demonstrating nonlinear optical properties are mostly carried out on non-flexible substrates and there is a huge demand for the fabrication of high performing flexible optoelectronic devices using new exotic materials due to their potential applications in wearable devices, communications, sensors, imaging etc. Here first time we integrate the thin films of TIs (Bi_2_Te_3_) with the flexible PET (polyethylene terephthalate) substrate and report the strong light absorption properties in these devices. Owing to small band gap material, evolving bulk and gapless surface state conduction, we observe high responsivity and detectivity at NIR (near infrared) wavelengths (39 A/W, 6.1 × 10^8^ Jones for 1064 nm and 58 A/W, 6.1 × 10^8^ Jones for 1550 nm). TIs based flexible devices show that photocurrent is linearly dependent on the incident laser power and applied bias voltage. Devices also show very fast response and decay times. Thus we believe that the superior optoelectronic properties reported here pave the way for making TIs based flexible optoelectronic devices.

## Introduction

Nowadays optoelectronic devices used in optical communications or infrared imaging demand high performing photodetection properties along with flexibility of the substrate. Next generations optoelectronic devices will be majorly based on the flexible substrate/devices and potential application includes touch screen and displays, biomedical sensors, artificial skins, solar cells, flexible displays etc. Present utmost requirement is to integrate novel/2D (two dimensional) materials into flexible substrate and plastic substrates are mostly preferred for making flexible devices due to low cost, light weight, shock resistance, flexibility and can be integrated with the semiconducting materials by depositing thin films either by physical or chemical methods. Recently 2D or layered materials like transition metal dichalcogenides (TMDs), perovskite nanowire array films, perovskite heterostructures (PHSs), methylammonium lead iodide (CH_3_NH_3_PBI_3_) perovskite, metal-selenide heterostructures, black phosphorous (bP)/MoS_2_ heterojunction, graphene, TIs, etc. have shown very exciting optoelectronic properties and promising materials for flexible devices^1–22^.

Topological insulators (TIs) possess metallic surface states with spin-momentum locked massless Dirac fermions. A thin layer of TI is predicated as a promising material for high performing optoelectronic devices ranging from terahertz (THz) to infrared detections and applications include high speed optical communications, terahertz lasers and photodetectors, surveillance, remote sensing, thermal detection, waveguide etc^23^. Hence TI based materials have emerged as an excellent material reported for broadband (UV to NIR)^24–29^, visible to mid infrared (3.8 μm)^30^, deep UV to MIR^3^ photodetections. TI is also reported as a transparent conductor for infrared wavelength^7,31^ which is better when compared with ITO (indium tin oxide) films that show poor performance during infrared sensing and imaging applications. Bi_2_Te_3_ is a narrow gap semiconducting material and also a TI with metallic surface state investigated experimentally^32–34^. The heterostructure of TIs and Si showed photodetection over a wide range from UV to THz^35^. It has been also observed that bismuth chalcogenide based TIs are very stable even at ambient conditions^36^ and show robust nature of Dirac fermions even at room temperature^37^. The unique Bi_2_Te_3_- single walled nanotube networks^38,39^ and thin films of Bi_2_Te_3_ on flexible polyimide substrates^40^ were used to make high performing flexible thermoelectric generators for energy harvesting applications. Previously thin films of TIs made from the nanosheets of Bi_2_Se_3_ and Bi_2_Te_3_ were integrated with the plastic substrates using colloidal nanoplates ink for investigating the optoelectronic properties^41^ but films were not studied for their NIR photodetector performances e.g. responsivity and detectivity.

Integrating TI with flexible substrate, the performance of the photodetector / photodetection may degrade and many optimizations are needed to find good photoconducting device using a flexible substrate which is a challenging task. The concentrated colloidal nanoparticle, nanosheets or ink solutions can be processed into topological insulator or semiconducting films on flexible substrate^42^. Such solutions or ink based methods are tricky in controlling the thickness of the film and often hampered with the grain size and boundary defects, poor contacts between the adjacent nanoparticles etc. Nanomaterials do show better response but far from technological applications and low cost production. Sputtered coated films find more advantages over controlling the thickness and uniform large area growth. Here we report optoelectronic performance of sputter deposited thin films of TI on the flexible PET substrate. Considering the thin film options and advantages for low cost device fabrication, these flexible substrates could be scaled up to a sensor array. The NIR responsivity observed in these devices is in the range of 30–60 A /W which is very competitive for technological applications.

## Results and discussion

**Figure 1:**
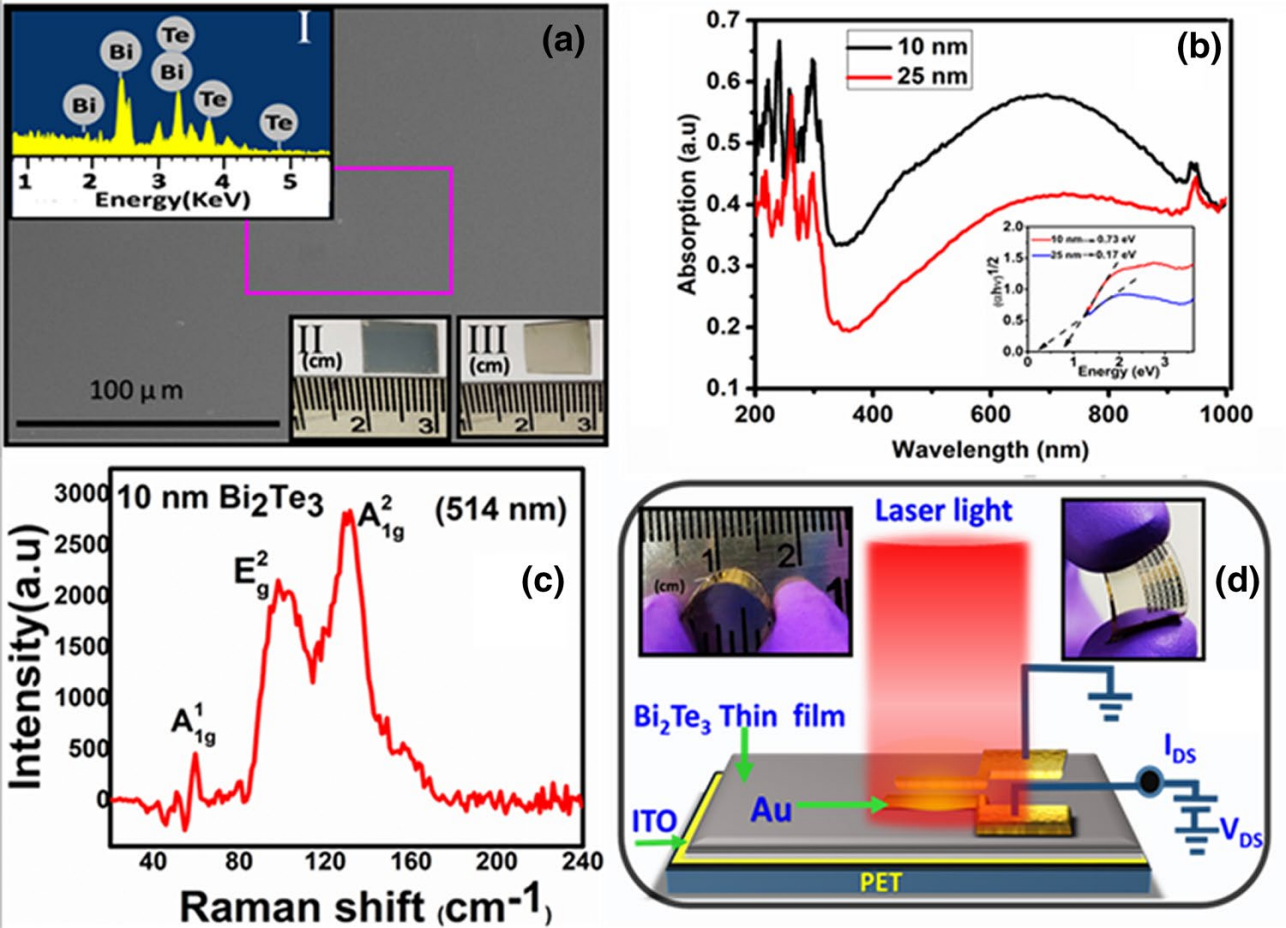
Characterization of flexible TIs thin film (Bi_2_Te_3_ on PET substrate) (**a**) FESEM image of the FTTF (flexible TIs thin film), inset I is the EDS data of the selected portion as shown in the figure. Inset II and III (right side corner) show the deposited FTTF with variation in thickness. (**b**) UV absorption spectra of FTTFs. The black and red curves represent UV absorption data for 10 nm and 25 thick films respectively. Inset represents the estimation of the bandgap. (**c**) Raman characterization of the film. (**d**) Schematics of the photodetector device and insets show the flexibility of the deposited film.

Figure 1a shows the FESEM image of TI thin film deposited on the flexible substrate (PET). Inset I in Fig. 1a displays the elemental analysis of the deposited film which was obtained using the energy dispersive spectroscopy. It shows the presence of bismuth and telluride and the percentage was found about Bi = 40.28 and Te = 59.72 which is very close to their atomic ratio percentage 2:3. The film was deposited on an area of about 10 × 10 mm^2^ as shown in the inset II and III of Fig. 1a. The appearance or colour of the film was found to be dependent on the thickness of the film which is expected because the films or nanosheets of topological insulators exhibit thickness dependent optical properties^23,43,44^. Figure 1b displays thickness dependent UV absorption spectroscopy for 10 nm (black curve) and 25 nm (red curve) Bi_2_Te_3_ films deposited and inset represents the estimation of band gap by using the Tauc method i.e. ∼ 0.17 eV was observed for 25 nm film. The Raman spectroscopy was used to know the quality of the film (Fig. 1c). We observed the optical phonon peaks, A^1^_1g_, (∼ 60 cm^−1^), E^2^_g_ (∼ 99 cm^−1^), and A^2^_1g_ (∼ 132 cm^−1^) when the film was excited at 514 nm wavelength. The previous work also observed the similar Raman peaks of the Bi_2_Te_3_ samples^45,46^. Figure 1d displays the schematics of the setup used for the optoelectronic / photocurrent/ photodetection studies. The light illumination area was more than the device area and insets in Fig. 1d show the bending of the device which was used for the photocurrent studies.

**Figure 2:**
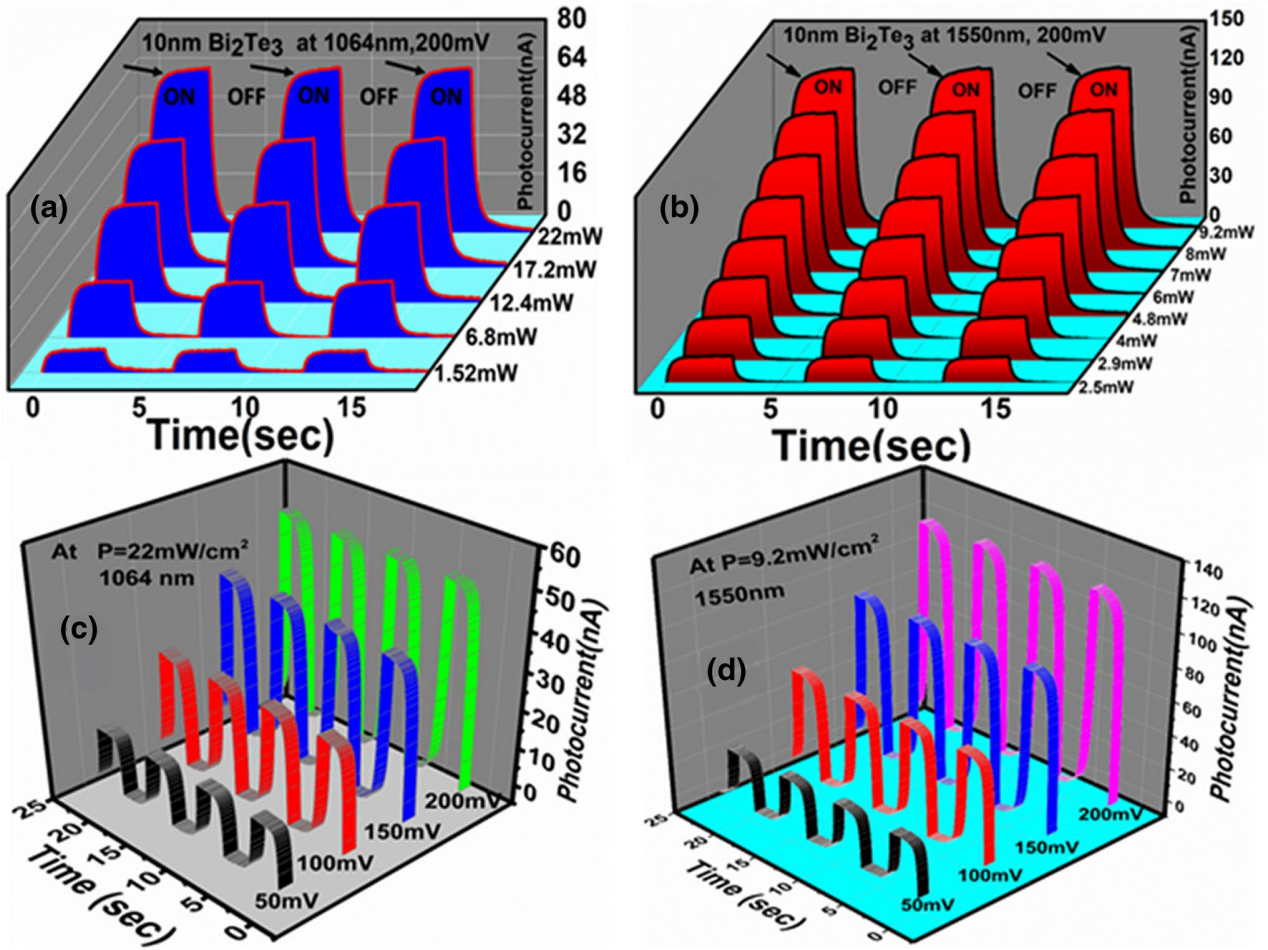
Photocurrent response of flexible TI thin film (**a**, **b** and **c**, **d**) power and bias dependent measurements under the illumination of 1064 nm (**a** and **c**) and 1550 nm (**b** and **d**) light respectively.

The optoelectronic response of the Bi_2_Te_3_ films deposited on the flexible substrates were thoroughly studied for different bias voltages and light illumination densities as shown in the Fig. 2. The near infrared response at constant bias 200 mV as a function of time for the wavelengths 1064 nm and 1550 nm is shown in the Fig. 2a,b respectively. The power of 1064 nm laser was tuned from 1.5 to 22 mW and the corresponding change in the device’s current was recorded. The photocurrent ${I}_{ph}$ was calculated as

1 $${I}_{ph}={I}_{light}-{I}_{dark}$$

where ${I}_{dark}$ is the dark current measured when the light illumination was off and ${I}_{light}$ is the measured current when the light was turned ON. The sudden rise and decay in the curves indicate the laser on and off states respectively and device clearly shows the response to the incident light. To know the rise and decay time constants, we used equation nos. 2 and 3 and the values were estimated as ∼ 190 ms and 180 ms for the wavelength 1064 nm respectively (Figure S1).

2 $${I}_{ph}={I}_{o}\left(1-{e}^{\frac{-t}{{\tau }_{r}}}\right)$$

3 $${I}_{ph}=A{ e}^{\frac{-t}{{\tau }_{d}}}$$

where ${I}_{o}$= saturated value of the photocurrent (shown by an arrow), ${\tau }_{r}$= rise time constant, ${\tau }_{d}$= decay time constant and $A$ is the fitting constant. The measurements were repeated for telecom wavelength (1550 nm) and data is shown in the Fig. 2b. Here the incident light illumination density was varied from 2.5 to 9.2 mW and the corresponding change in device’s current was recorded as a function of time. All the recorded curves display clear response to the incident light. The response and decay time constants for 1550 nm were estimated from the Eqs. 2 and 3 and the fitted curves can be found in the Figure S2. The increase in laser power shows the increase in the photocurrent and such effect was also studied earlier^47^. The photocurrent also depends on the applied voltage because the transit time (${T}_{t}$) of the carriers through the device channel is given by

4 $${T}_{t}={l}^{2}/\mu V$$

where $V=\mathrm{applied\; bias\; voltage},$ *l* = channel length and $\mu$= carrier mobility. The bias voltage dependent photocurrent was studied for both the wavelengths (1064 nm and 1550 nm) and plotted in the Fig. 2c,d respectively. The bias voltage was tuned from 50 to 200 mV and corresponding change in current as a function of time was measured. Note that the Bi_2_Te_3_ thin film on flexible substrate clearly responds to different applied voltages as well as incident laser powers. The photocurrent as a function of applied bias voltage or light power is plotted in the Figure S3 and the linear increase in photocurrent was noticed.

**Figure 3:**
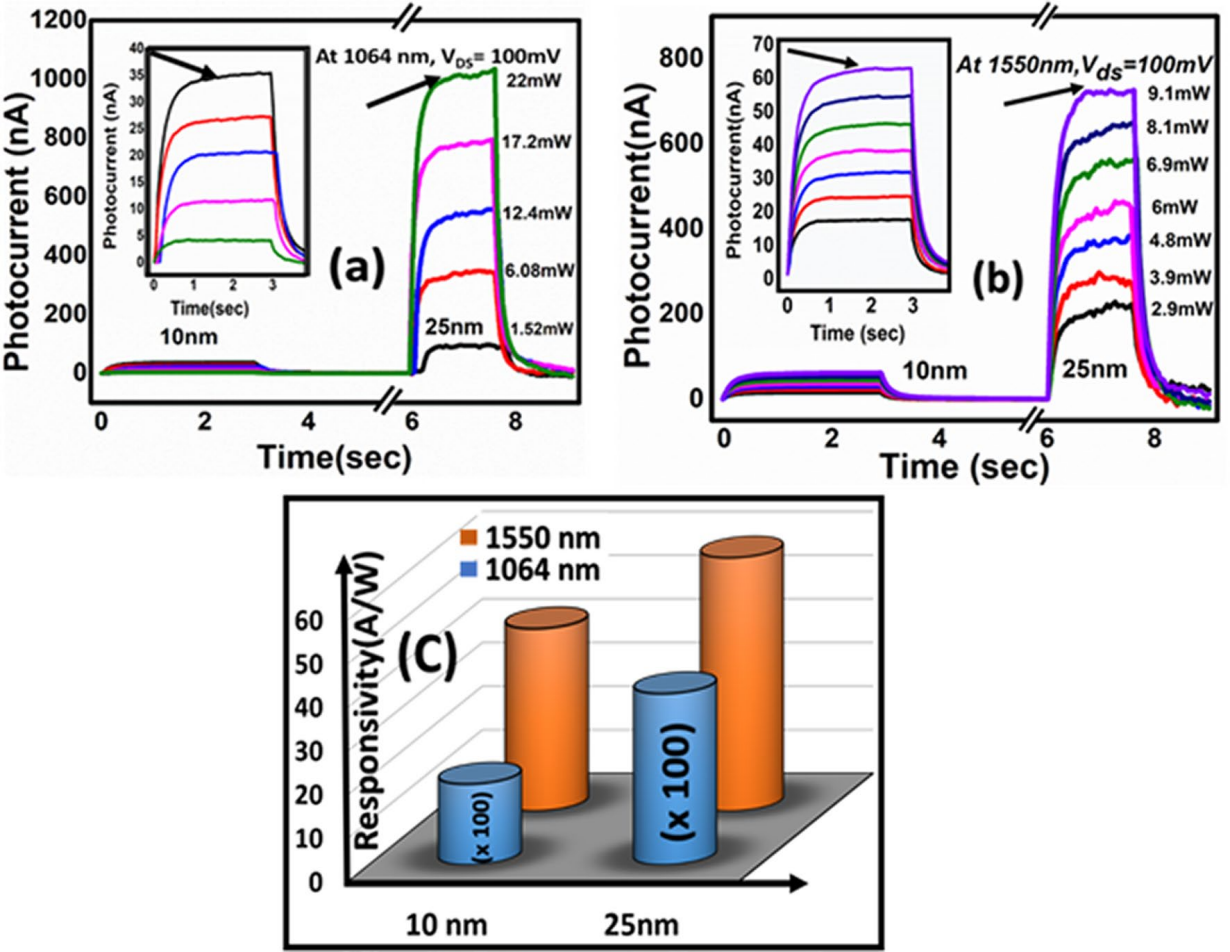
Thickness dependent photocurrent measurements. (**a**,**b**) Bias voltage dependent photocurrent under the illumination of 1064 and 1550 nm wavelengths. (**c**) Thickness dependent comparison of responsivity values.

Since the performance of the photodetection using TI films depends on the thickness of the film^23,48^ , we report the photoresponse of the flexible thin film of Bi_2_Te_3_ studied for different thicknesses (10 and 25 nm) which is shown in the Fig. 3. Both the films clearly show (Fig. 3a,b) sudden increase and decrease in the current value indicating the laser ON and OFF states respectively. Compared to 25 nm thick films, magnitude of photocurrent in 10 nm thick film was found ~ 25 times less under the illumination of 1064 nm (Fig. 3a). Similarly, about 10 times less photocurrent was observed in 10 nm film when it was irradiated with the telecom wavelength 1550 nm (Fig. 3b). It indicates that more carriers are excited in the thick films which could be due to the bulk contribution. The earlier literature suggests that in topological insulator material there is a possibility of photocurrent contributions originating from the bulk of the sample as well as from the surface states also^47,49,50^. Thickness dependent optical properties were studied theoretically and decrease in optical conductivity was observed with decrease in thickness of the film^48^. Compared with the 25 nm thick TI film, bulk subbands are very less in 10 nm film which indicates that the thickness approaches to the quantum size effects. At this critical thickness, the no. of interband transitions could be less in the bulk states which may result in the decrease in photoresponsivity of 10 nm thin film. Insets in Fig. 3a,b represent the photocurrent data for 10 nm thin film which is not visible when we compared with the 25 nm film. The responsivity was estimated by using the relation

5 $${R}_{\mathrm{ph}}=\frac{{I}_{ph}}{{P}_{d} \times A}$$

where ${I}_{ph}$,${P}_{d}$ and $A$ are photocurrent, power density and effective device area respectively^51^. The responsivity of about 58 A/W was observed for the 1550 nm wavelength which is very competitive for thin film based flexible devices when compared with the other TIs based thin films photodetectors (Table 1) and we have observed better responsivity for the telecom wavelength 1550 nm (Fig. 3c). Further the detectivity (*D*) and external quantum efficiency (*EQE*) were estimated from the following equations.

6 $$D (Detectivity)= \frac{{R}_{ph}\sqrt{A}}{\sqrt{2q{I}_{D}}}$$

7 $$EQE =\frac{1240{R}_{\lambda }}{\lambda }$$

where *I*_*D*_ = dark current, *q* = electrons charge, *R*_*ph*_ = responsivity, *A* = effective device area, *R*_*λ*_ = responsivity at specific wavelength and *λ* = wavelength.

**Table 1:** Thin films of TIs based flexible and non-flexible photodetectors.

Material	λ (nm)	R (A/W)	Detectivity (jones)	Gain/EQE (%)	Raise time (ms)	Decay time (ms)
2D Bi_2_Te_3_–SnS–Bi_2_Te_3_^24^ (Thickness < 10 nm)	370	115	4.1 × 10^11^	3.9 × 10^4^	–	–
Bi_2_Te_3_/ pentacene on mica^53^ (Thickness 100 nm)	450–3500	14.89	2840	–	1.89	2.47
Bi_2_Te_3_ thin film on mica^41^ (Thickness 100 nm)	450–4850	2.82	601	–	8.7	19.3
Bi_2_Se_3_/Si Heterostructure^54^	808	24.28	4.39 × 10^12^	–	0.0025	0.0055
Sb_2_Te_3_ film^55^ (Thickness 50 nm)	980	21.7	1.22 × 10^11^	27.4	238.7 × 10^3^	203.5 × 10^3^
Polycrystalline Bi_2_Te_3_ film/Si^35^ (Thickness 100 nm)	635	1	2.5 × 10^11^	–	100	100
25 nm Bi_2_Te_3_ film on flexible (this work)	1064	39.217	6.123 × 10^8^	45.896%	110	95
	1550	58.006	6.251 × 10^8^	46.46%	132	78

**Figure 4:**
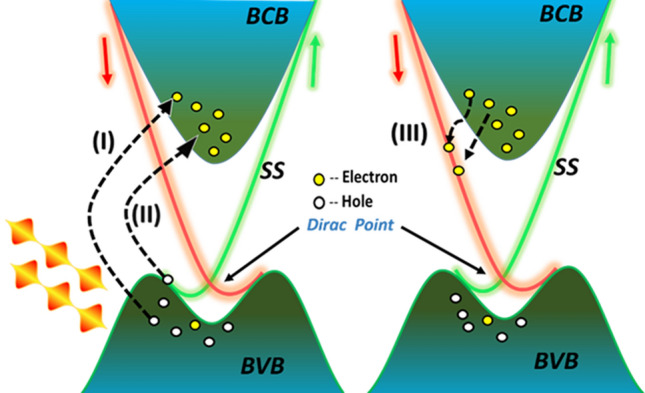
Schematic of energy bands and optical excitation of carriers in TIs thin film.

The high photoresponse observed for the NIR wavelengths could be due to interband transitions happening in bulk and surface states from the valance band to bulk and surface state in the conduction band indicating the possibility of electron hole pair generation in bulk and surface states (Fig. 4). Such optical transitions were observed theoretically for the high radiation frequency > 300 meV^48^. Overall strong optical absorption process in TIs films involves transitions originating from the interbands, intrabands and surface states. There is also possibility of excited carriers drifting towards the surface state dominated conducting channel and excitation with energy more than the band gap of the material could lead carriers to the surface state filling behavior from a metastable population in the bulk conduction band^52^. This gives rise in the photocurrent of the device under a voltage bias measurements. Note that the role of topological surface states discussed here for the observation of high photo response is hypothetical in nature and more experimental results are necessary for thorough understanding of it. Further for practical applications along with flexibility and good NIR responsivity, it is equally important to have a photosensitivity of the material over a broadspectral range. Apart from the NIR photodetection, these flexible TI films also show photoresponse under the illumination of visible light and data is shown in the Figure S4. The clear increase/decrease in the current of the devices was observed in presence of light ON and OFF conditions at a constant bias voltage. The rise and decay times were observed in the range of ∼ 100 ms for visible wavelength 532 nm. The response time depends on the crystalline quality of the film and it can be improved further if the films are deposited using techniques like MBE (molecular beam epitaxy). The performance of the thin film was found working well after 8 months storage at ambient conditions which indicate the robustness of the material (Figure S5).

It is important to integrate TI based materials with flexible substrate because TIs possess metallic conducting surface states and massless Dirac fermions are present on these TSS which are protected by time reversal symmetry and show nonlinear optoelectronic properties detecting light over a broad spectral range^23^. The helical nature of TSS suppresses back scattering and dissipation less electron transport is predicated in these materials. Other hand PET is a very common substrate and one can easily deposit TI films by using sputtering technique on them. Here we demonstrated the successful integration of Bi_2_Te_3_ films with PET and believe that other TI films can be integrated by this way. We have observed highly efficient NIR photodetection in flexible Bi_2_Te_3_ thin films under the illumination 1064 and 1550 nm wavelengths. The Bi_2_Te_3_ films deposited on the Si substrate show responsivity of about 3.64 × 10^–3^ A/W and 3.32 × 10^-2^A/W for NIR wavelengths 1064 nm and 1550 nm respectively^56^. Here our devices made using flexible substrates show more than three orders improvements in the responsivity values. The high performance of Bi_2_Te_3_ films was compared with the other flexible TIs based thin film devices and is shown in the Table 1 which clearly demonstrates the competitiveness of flexible devices studied here.

## Conclusion

The high performing flexible phototodetectors made from the topological based material are presented here which operate efficiently under the NIR as well as visible wavelengths. A high responsivity was observed for telecom wavelength which is very competitive compared to the available thin film based flexible photodetectors as shown in the Table1. The thickness dependent response in the photocurrent was observed which might indicates the contribution of the bulk transport effect in the photocurrent. Overall TI films deposited on flexible PET substrates can be used for the broad spectral photodetection with high responsivity for the telecom wavelength and may be considered as a suitable material for fabricating the wearable optoelectronic devices.

## Methods

### Experimental section

*Sputtering technique* was used to deposit the Bi_2_Te_3_ thin films. A commercially available high purity Bi_2_Te_3_ target (99.99% purity) was used as a source in the sputtering system. Before being loaded into the deposition chamber, the PET substrates were sequentially washed by acetone, isopropanol, methanol, deionized water and treated with oxygen plasma for 5 min to remove both the organic and inorganic contaminations. A thin layer of Bi_2_Te_3_ film (∼ 10/25 nm) was deposited on these cleaned substrates using sputtering technique (base pressure > 5 × 10^–7^ mbar, deposition pressure 3 × 10^-3^ mbar, argon flow ∼ 20 sccm). The thickness of the film was optimized and very thin layer of Bi_2_Te_3_ was deposited. During deposition substrate temperature was kept at room temperature. For optoelectrical characterization, the electrical pads (∼ 110 nm) were made using gold (100 nm) and titanium (10 nm) layers on these sputtered Bi_2_Te_3_ films through shadow masking procedure. The shadow masks were custom designed and made by Tecan Ltd, UK. For thickness-dependent measurement, the thin films with thickness varying between 5 and 50 nm were deposited. *The FESEM characterization* (Zeiss, Auriga) was performed to know the surface morphology of the thin films and EDS (energy dispersive spectroscopy) was used for elemental analysis of the thin films. The EDS spectrum detecting peaks of Bi and Te elements is shown in the manuscript and atomic weight percentage was found about Bi ≈ 40.28 and Te = 59.72 respectively, which indicates the ratio between Bi and Te is nearly 2:3. (e.g., Te–Bi–Te–Bi–Te), which corresponds to the Bi_2_Te_3_ formula. *The Raman spectroscopy* was used to know the quality of Bi_2_Te_3_ thin films and the spectra were recorded at 514 nm excitation. Three optical phonon peaks, A^1^_1g_ (∼ 60 cm^−1^), E^2^_g_ (∼ 99 cm^−1^), and A^2^_1g_ (∼ 132 cm^−1^) were observed and identified. These peaks were found very close to previous measured and assigned Raman peaks of the Bi_2_Te_3_ samples. *The UV–Visible measurements* were carried out at room temperature and under ambient conditions using a UV–Vis Spectrometer (Model No.: AvaLight-DH-S-BAL) and wavelength range was selected 200–1100 nm. The substantial light absorption was observed throughout the visible region. The band gap of film was calculated with the help of transmission spectra, using the Tauc method. *The electric measurements were* carried out at room temperature under ambient condition using a probe station (Cascade Microtech with shield enclosure) accompanied by dual source meter Keithley-2634B and laser sources 532, 1064 and 1550 nm.

## Supplementary Information

Supplementary Figures.

## Data Availability

All experimental data required to evaluate and interpret the conclusions are present in the main manuscript or supplementary materials file. Additional data or information related to this paper may be requested from the corresponding author.
