# Supplementary material for: High performing flexible optoelectronic devices using thin films of topological insulator
Source: Sci Rep. 2021 Jan 12;11:832. doi: 10.1038/s41598-020-80738-8 (PMC7804467; doi:10.1038/s41598-020-80738-8)
Supplement: Supplementary file 1 — Supplementary Figures. [file 41598_2020_80738_MOESM1_ESM.pdf]

Supplementary Information

High performing flexible optoelectronic devices using thin films of topological insulator

Animesh Pandey[1,2], Reena Yadav[1,2], Mandeep Kaur[2], Preetam Singh[1,2], Anurag Gupta[1,2] and Sudhir Husale[1,2]*

[1] *Academy of Scientific and Innovative Research (AcSIR), National Physical Laboratory, Council of Scientific and Industrial Research, Dr. K. S Krishnan Road, New Delhi-110012, India.*

[2] *National Physical Laboratory, Council of Scientific and Industrial Research, Dr. K. S Krishnan Road, New Delhi-110012, India.*

**E-mail: husalesc@nplindia.org*

Supplementary Information includes:

1. Fig S1. Rise and decay time fits at constant bias volt 100 mV. Bias voltage dependent rise and decay times for the 1064 nm laser light illumination.

2. Fig S2, Rise and decay time fits at constant bias volt 100 mV. Bias voltage dependent rise and decay times for the 1550 nm laser light illumination.

3. Fig S3, The relationship between photo-current versus applied power/bias voltage

4. Fig S4. The response of the Bi_2Te_3 device measured under visible light 532 nm

5. Fig S5. Robust photodetection properties of Bi_2Te_3 thin films

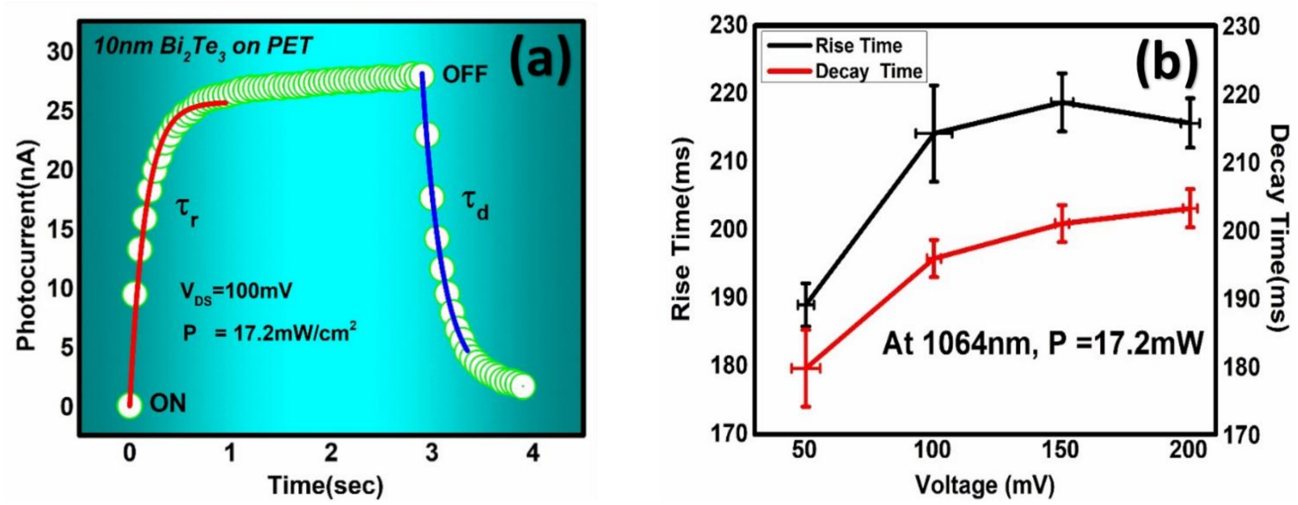

Fig S1 a) shows rise and decay time fits at constant bias volt 100 mV b) bias voltage dependent rise and decay times for the 1064 nm laser light illumination.

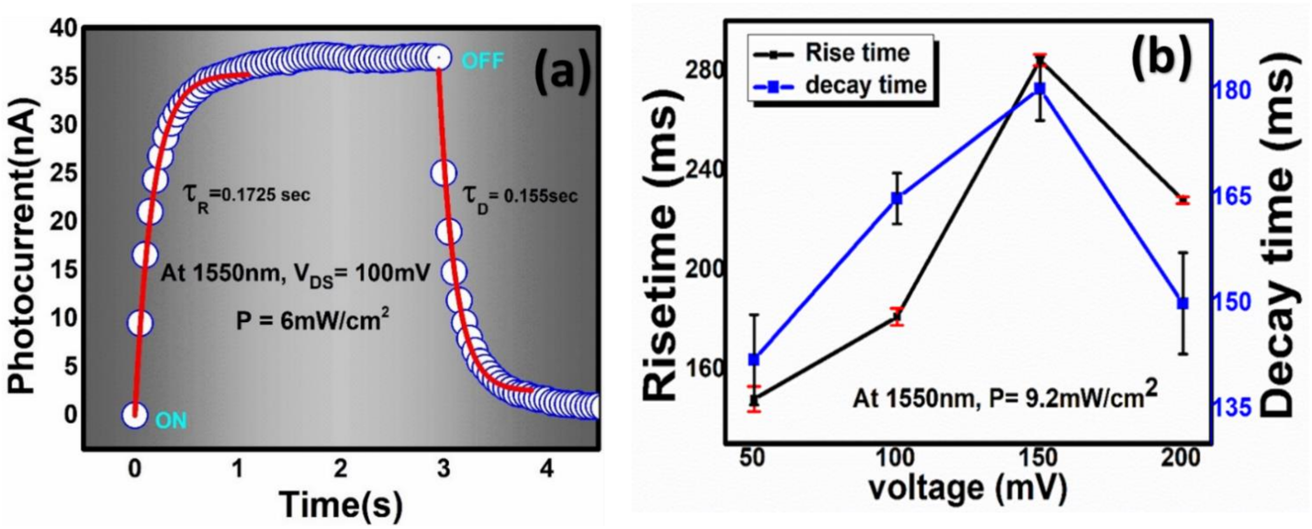

Fig S2 a) shows rise and decay time fits at constant bias volt 100 mV b) bias voltage dependent rise and decay times for the 1550 nm laser light illumination.

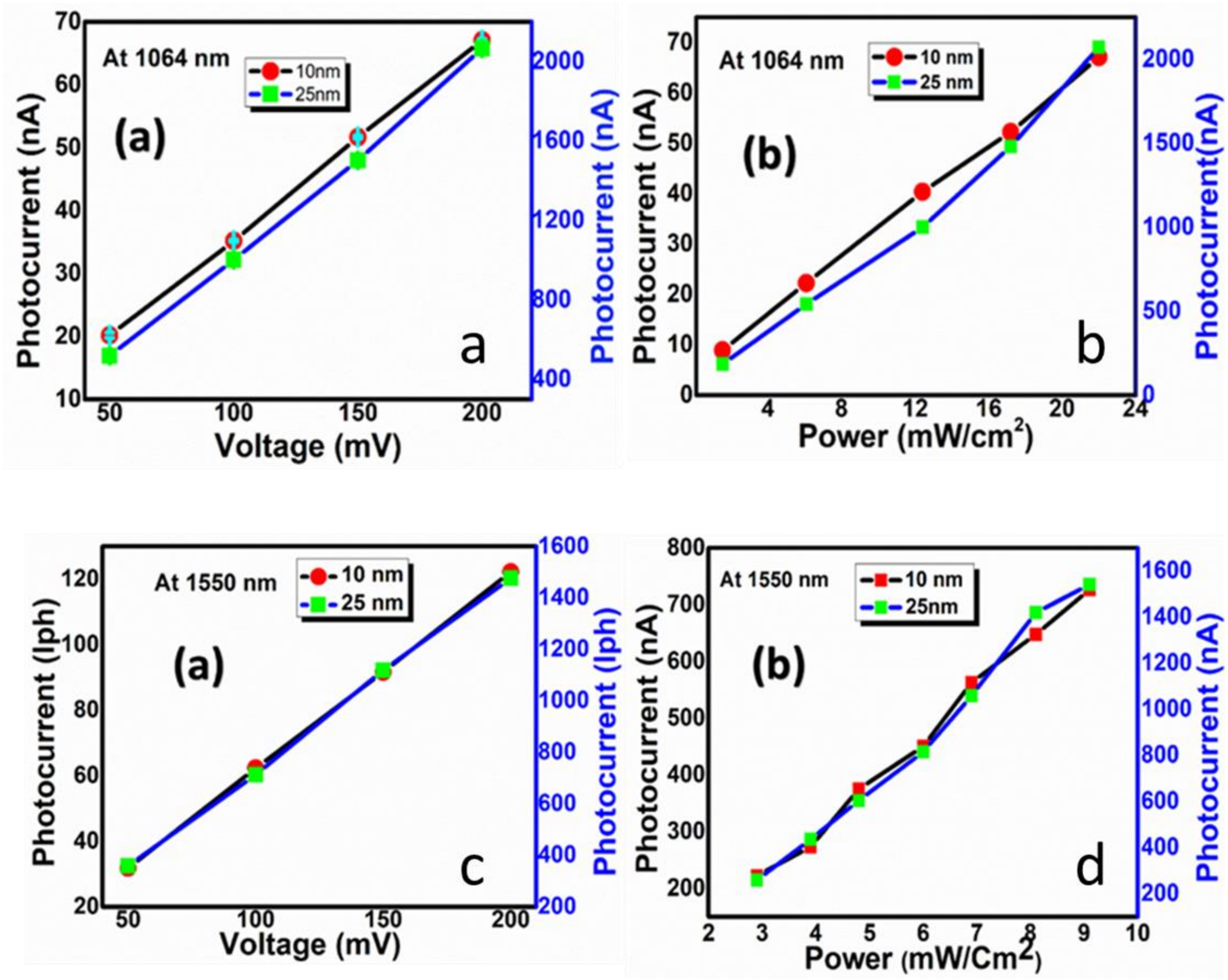

Fig S3, The relationship between photo-current versus applied power/bias voltage: **Fig S3 (a)** shows the thickness dependent photocurrent (10 and 25 nm) at 1064 nm wavelength as a function of bias voltage. **Fig S3 (b)** represents the thickness dependent photocurrent at 1064 nm wavelength as a function of laser power density. **Fig S3 (c)** shows thickness dependent photocurrent at 1550 nm wavelength as a function of bias voltage and **Fig S3 (d)** represents the thickness dependent photocurrent at 1550 nm wavelength as a function of laser power density.

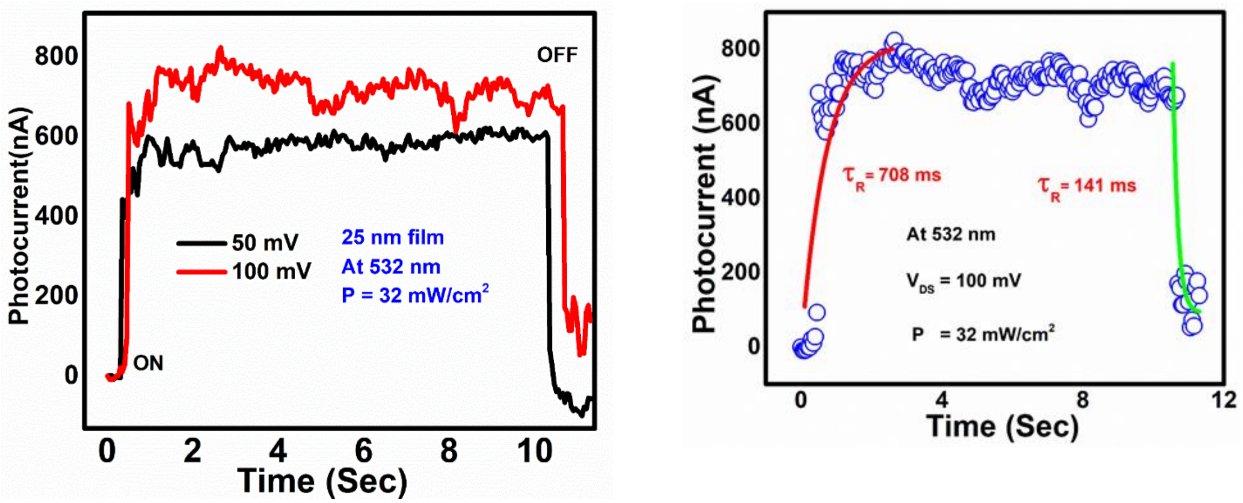

Fig S4 shows the response of the Bi_2Te_3 device measured under visible light 532 nm

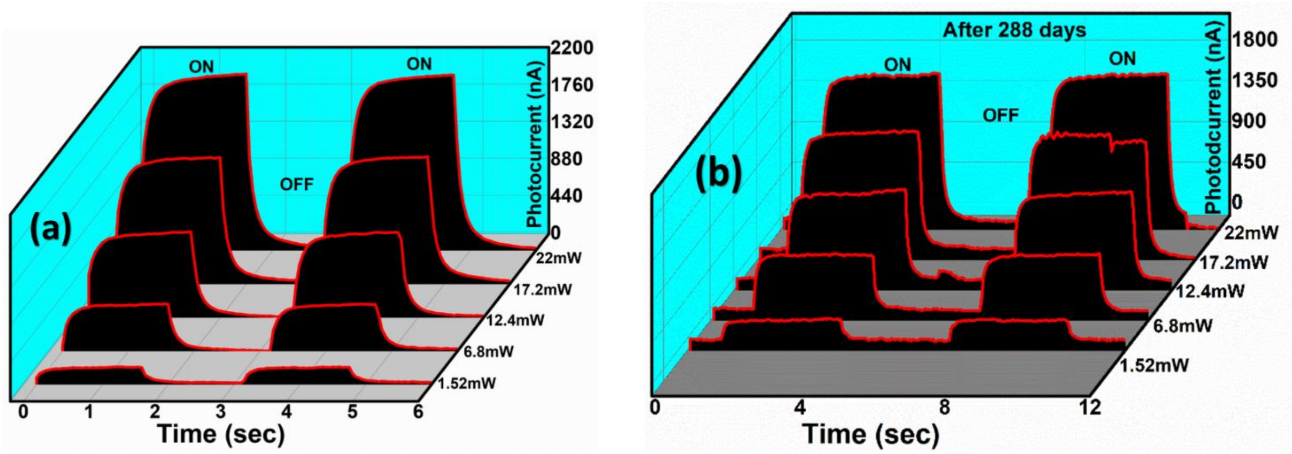

Fig S5 a) shows the bias voltage and time dependent photocurrent response of the Bi_2Te_3 device measured under NIR light 1064 nm, b) measurements performed after 288 days (Note that many devices (array like) were fabricated on the flexible thin film. Randomly few of them were selected, measured and were found working even after 288 days).
